# Valuing breastfeeding: a qualitative study of women’s experiences of a financial incentive scheme for breastfeeding

**DOI:** 10.1186/s12884-017-1651-7

**Published:** 2018-01-08

**Authors:** Maxine Johnson, Barbara Whelan, Clare Relton, Kate Thomas, Mark Strong, Elaine Scott, Mary J. Renfrew

**Affiliations:** 1 School of Health and Related Research (ScHARR), University of Sheffield, Sheffield, UK; 2 Mother and Infant Research Unit, School of Nursing and Health Sciences, University of Dundee, Dundee, UK

**Keywords:** Financial incentives, Breastfeeding, Qualitative

## Abstract

**Background:**

A cluster randomised controlled trial of a financial incentive for breastfeeding conducted in areas with low breastfeeding rates in the UK reported a statistically significant increase in breastfeeding at 6–8 weeks. In this paper we report an analysis of interviews with women eligible for the scheme, exploring their experiences and perceptions of the scheme and its impact on breastfeeding to support the interpretation of the results of the trial.

**Methods:**

Semi-structured interviews were carried out with 35 women eligible for the scheme during the feasibility and trial stages. All interviews were recorded and verbatim transcripts analysed using a Framework Analysis approach.

**Results:**

Women reported that their decisions about infant feeding were influenced by the behaviours and beliefs of their family and friends, socio-cultural norms and by health and practical considerations.

They were generally positive about the scheme, and felt valued for the effort involved in breastfeeding. The vouchers were frequently described as a reward, a bonus and something to look forward to, and helping women keep going with their breastfeeding. They were often perceived as compensation for the difficulties women encountered during breastfeeding. The scheme was not thought to make a difference to mothers who were strongly against breastfeeding. However, women did believe the scheme would help normalise breastfeeding, influence those who were undecided and help women to keep going with breastfeeding and reach key milestones e.g. 6 weeks or 3 months.

**Conclusions:**

The scheme was acceptable to women, who perceived it as rewarding and valuing them for breastfeeding. Women reported that the scheme could raise awareness of breastfeeding and encourage its normalisation. This provides a possible mechanism of action to explain the results of the trial.

**Trial registration:**

The trial is registered with the ISRCTN registry, number 44898617, https://www.isrctn.com

**Electronic supplementary material:**

The online version of this article (10.1186/s12884-017-1651-7) contains supplementary material, which is available to authorized users.

## Background

The World Health Organisation recommends exclusive breastfeeding for the first 6 months of an infant’s life, followed by continued breastfeeding alongside solid foods until at least 2 years of age [1]. There are a range of risks associated with not breastfeeding [2], including increased morbidity [3] and related healthcare and treatment costs [4]. Despite all four UK Departments of Health endorsing the WHO recommendations, and multiple initiatives to improve breastfeeding rates, the average 6–8 week prevalence in England during 2015/2016 was below 46% [5].

Financial incentives have previously been used in public health to motivate behaviour change, for example smoking cessation in pregnancy [6]. One small-scale, peer-support led, gift based incentive for breastfeeding was generally positively received by participating women and peer supporters [7]. However, authors of a systematic review of non-financial incentives to improve breastfeeding rates were not able to determine the overall effectiveness of incentives due to the heterogeneity of the studies identified [8].

The Nourishing Start for Health (NOSH) research project developed and tested the effectiveness of a financial incentive scheme in the form of vouchers which were exchangeable at supermarkets and other retail shops with no restriction on allowable purchases. The scheme was initially developed with midwives, health visitors, healthcare commissioners, breastfeeding peer support workers and local women [9], resulting in offered vouchers worth £200 paid in five £40 instalments at time points based on infant age: 2 days, 10 days, 6–8 weeks, 3 months and 6 months. Receipt of vouchers was conditional on mothers and healthcare professionals (HCPs) signing and countersigning a form stating that exclusive or partial breastfeeding was taking place. HCPs had the option of confidentially notifying the research team if they had a concern that an infant whose mother was claiming was not receiving breast milk, without those claims being jeopardised.

Women and HCPs were interviewed at each of the three stages of the NOSH project. Responses from the first stage (development) were used to inform the study design [9] which was tested in the second (feasibility) stage [10]. The purpose of this paper is to help interpret the results of the full trial (details [11] and findings [12] are published elsewhere). The trial reported a significant increase in breastfeeding at 6–8 weeks for those clusters where the NOSH Scheme was offered [12]. Findings from interviews with HCPs during the trial stage have been submitted for publication (Whelan B, Relton C, Johnson M, Strong M, Thomas K, Umney D, Renfrew MJ. Healthcare professionals' experiences of an area level conditional cash transfer scheme for breastfeeding. Forthcoming). This paper reports on interviews with women eligible for participation in the second and third stages of the project.

## Methods

### Aim, design and setting

Interviews were conducted in order to identify, through women’s reports, influences on infant feeding decisions, (dis) satisfaction with (non) participation in the scheme, and the ways in which participation might impact on breastfeeding. A qualitative design was used for this part of the study, utilising semi-structured interviews, to provide rich data rather than responses to, for example, a survey tool. Pilot interview schedules were developed and shared with the research team for feedback. The final versions were piloted and adapted following discussion with the team.

We report qualitative data collected from single interviews with 35 women during the pre-trial feasibility and the cluster randomised controlled trial stages (see Table 1 for participant characteristics). All women lived in electoral wards with breastfeeding rates < 40% at 6–8 weeks (the primary endpoint for the trial). The mean area-level deprivation scores were higher (more deprived) than the average mean for England [12]. The pre-trial feasibility stage assessed the acceptability and deliverability of the NOSH scheme in three areas of Sheffield, Rotherham (South Yorkshire) and Chesterfield (North Derbyshire). The cluster trial stage was conducted across a wider area that also included Doncaster, Bassetlaw and an extended region of North Derbyshire. The same financial incentive scheme was offered during both the feasibility and trial stages thus we considered it appropriate to analyse both sets of data together.

**Table 1:** Characteristics of women interviewed

Age	(n)	Previous children	
18–24	6	Yes	19
25–29	8	No	16
30–34	15		
35–40	6	Applied to participate in NOSH	
		Yes	31
District		No	4
Sheffield	5		
Rotherham	12	No of claims for NOSH Scheme voucher at time of interview	
Chesterfield	5	No of claims	No of women
N. Derbyshire	5	0	7 (5 did not participate)
Bassetlaw	6	1	2
Doncaster	2	2	2
		3	9
Ethnicity		4	5
White British	33	5	10 (this includes double claim for twins)
White other	2		

### Recruitment and participants

Women were recruited for interview by health visitors either by postal invitation or in health visitor clinics in Children’s centres. Researchers developed a sampling frame in order to purposively sample women with a mix of characteristics (for example, age, electoral ward) who were willing to be interviewed, This included women who were eligible for the scheme regardless of whether or not they had or had not participated in the scheme. Women who expressed an interest in being interviewed were sent an information sheet about the study and contacted by the researchers (BW, PvC and MJ) by telephone or e-mail to organise a telephone or in-person interview. Women were given the opportunity to ask questions about the study prior to signing a consent form.

The ages of the interviewees ranged between 18 and 40, and all but two (who described themselves as British Other) described themselves as British White. Just over half of the women were feeding their first baby. Five interviewees had not participated in the NOSH scheme, or had applied but not claimed vouchers because they did not breastfeed. A further two women had not claimed vouchers by the time they were interviewed. The remaining interviewees had claimed between one and five vouchers prior to interview.

### Data collection

All individual and group interviews were carried out by BW, MJ or PvC between 2015 and 2016, prior to the results of the trial being known. All three interviewers were female researchers with PhD qualifications and experience of carrying out qualitative research. Two had health visiting and nursing backgrounds and two had personal experience of infant feeding. Interview schedules were developed for women participating and not participating in the scheme by the study team and piloted during the first interviews with participants and non-participants of the feasibility phase (see Additional file 1).

Women mainly took part in one individual interview each (*n* = 33), though one group interview was also carried out (*n* = 2). Interviews were carried out in women’s homes and lasted between 30 and 70 min, depending on the participants’ available time. Interviews were audio-recorded and transcribed verbatim with written consent from the participants. Field notes were made following interviews of any details that might help with transcribing or analysis, for example, interruptions by babies or small children. We stopped making new participant contacts when no new accounts were emerging in interviews.

### Data analysis

Analysis of the data was carried out using the Framework approach [13], a method which was developed for systematically analysing qualitative data. The approach consists of five phases including familiarisation with data, the construction of a thematic framework, indexing the data and reviewing and summarising data. An initial framework was developed by BW and MJ following independent reading and joint discussion of the data. Themes were shared and discussed with CR and KJT prior to summarising in the Framework matrices (see Additional file 2).

At this point we (BW MJ CR KJT) knew the results of the NOSH trial, and were particularly interested in answering the question ‘How did the intervention increase breastfeeding rates?’ This article focusses on the themes from the data that relate to this question. A narrative structure (presented in the following section) was developed that included the final themes. NVivo 10 [14] software was used to enable data organisation and retrieval. The findings were not shared with participants as they represent an interpretation of the synthesis of women’s views and do not necessarily reflect the views of each participant [15].

## Results

In this section we present themes identified from analysed data that help to interpret the NOSH trial results and provide explanations for intervention mechanisms. The themes represent women’s experiences of infant feeding, participating in the NOSH scheme, and impact of the NOSH scheme.

### Deciding whether or not to breast feed

When asked about their initial infant feeding plans, women spoke of the influences that informed them, and how their initial plans developed or changed over time. Only one interviewee openly stated that she was influenced by the prospect of receiving vouchers. Rather, a range of influences were described including health-related information, practicalities such as preparing bottles, support from peers and health care professionals and their own past experiences.

#### Family and friends

Women reported being influenced by the attitudes and actions of their family and friends and the prevailing culture in their home area or country of origin. For some women, breastfeeding was the norm among their family and peers, whilst for others formula feeding was presented as the norm or the easier option, and breastfeeding as “*weird*” or “*disgusting*”,

“*I can think of two friends who think it’s a bit weird and disgusting and why would you get your breasts out and let your baby suck*…” (F17)

Other women reported times when family members were quick to suggest turning to formula feeding when they were struggling to breastfeed. In some cases they were able to provide reasons to override suggestions to stop breastfeeding:

“*But, I had a lot of people say to me, like family members, why don’t you just give her a bottle, you know even my other half, who was supportive when he saw me really upset and struggling he, why don’t you just give her a bottle, and that wasn’t the answer, you know the answer, he was, it wasn’t just, it’s not just about feeding, it’s about that bonding, and me feeling that’s what I wanted to give her*” (T7)

There were suggestions that social norms have oscillated through generations, with younger (and some middle aged) women now holding less positive attitudes to breastfeeding:

“*breastfeeding is kind of like, it died out didn’t it, I know I weren’t breastfed. And it’s kind of come full circle again and we’re trying to get it back in, we’re realising what a positive thing it is but I think people of a certain generation don’t kind of see the benefits of it*” (T8)

#### Past experience

Another important influence on women’s feeding decisions was their own previous experience. Breastfeeding was reported to be a greater challenge with first born babies due to an initial lack of knowledge, skill and confidence. Many of those who had previous experience of breastfeeding and made the decision to breastfeed with subsequent births, reporting these experiences as more relaxed. Women were either put off from breastfeeding by difficult experiences or more determined to breastfeed, feeling that they had somehow failed the first time:

“*when I had her I was more determined, because I think that’s when my baby blues set in when I’d finished feeding him because I knew it was my fault, I’d messed up*” (T5)

Reported negative past experiences also included lack of support to breastfeed in public spaces:

“*I changed me mind when I got kicked out of* *[restaurant name]* *for breastfeeding me daughter yeah cause someone complained and they told me to stop or get out so I got up and walked out so that put me off so I didn’t do it after that*” (F10)

This particular experience influenced the decision not to breastfeed her second child and not to apply for the NOSH scheme.

#### Health care professionals

Many women discussed how midwives, health visitors and infant feeding support workers encouraged breastfeeding from pregnancy onward. Such encouragement could sway the decision to breastfeed, and discussions about the NOSH scheme may have contributed to women’s perceptions of breastfeeding regardless of the financial incentive. Conversely, over-encouragement to breastfeed could foster annoyance:

*“Oh gosh yeah, all way through my pregnancy, every time I saw the midwife they discussed it….it’s just like you think (sigh) for goodness sake, I’ve made my own decision, let me, at the end of the day it’s my choice so let me make that choice.*” (F13)

Support from HCPs to initiate (or continue) breastfeeding post birth was inconsistent across different women’s reported experiences and sometimes women reported less support from hospital staff (often attributed to busy wards) than from community midwives and health visitors.

#### Changing from initial decision

Occasionally, women reported changing their decision about how to feed their baby after the birth. These changes were evident for both breastfeeding and formula feeding women. For example, one woman who did not breastfeed her first baby and did not intend to do so for her second, put her baby to the breast once at home:

*“I bottle fed him for the first two days then that night, I don’t know what it was I think it was just, I picked him up and I thought ‘your boobs feel like they need emptying anyway don’t they’, put him to me breast and I thought I’ll breastfeed him tonight and that were it then”* (F5)

She subsequently applied for the scheme and received vouchers, reporting that although this felt rewarding, it did not influence her decision to change. Another woman reported changing to breastfeeding because of the inconvenience of preparing bottles and formula.

Decisions to breastfeed were also subject to change following the birth. Reasons for such change were that the actual experience of breastfeeding did not match expectations. For example, one woman felt less positive toward breastfeeding in practice than she had during pregnancy. For another woman, insufficient milk was produced to nourish the baby, which led her to report that the decision not to breastfeed had been made against her will:

“*Well I wasn’t, you know it wasn’t my, it really wasn’t my decision, it’s my body you know made the decision for me but like I say I wanted to do it, that was the plan..*” (T4)

Whilst disappointment was reported when expectations changed, many women also showed awareness, usually from past experience, that their pre-birth feeding plans to breastfeed can change due to a number of unpredictable factors. In practice the factors reported for stopping breastfeeding included traumatic or premature birth, the baby not latching on, uncertainty regarding how much milk the baby was receiving, feeding becoming too frequent, infection and cracked skin, hospitalisation of mother or baby and perceived lack of support.

### Experiences of the NOSH scheme

#### Participants in the scheme

Participating women interviewed were generally positive about their experience of the process of applying to the NOSH scheme, claiming vouchers and their participation in the scheme. The following quotation is a typical response:

“*When I got the information booklet it was very straightforward after that. It explained everything to me so I knew, sort of each stage and then my health visitor filled in the rest but if I had any questions when she came out, she asked about it every time… how I was getting on and about it, so yeah, it’s very, very clear to us*” (T11)

Women reported spending their vouchers on the weekly shopping, food, nappies, and children’s clothes.

#### Non-participants in the scheme

Seven interviewees did not participate in the scheme. There were several reasons for not participating. One did not apply because she felt too busy at the time of the birth:

*“It was just that busy and that hectic that I never would have never even thought to get the paper work out or fill any information in”* (F15).

Two expressed uncertainty about the criteria for claiming vouchers, for example, for mixed feeding or for only 2 or 10 days of breastfeeding. Three women expressed their frustration about not being able to breastfeed and one of these reported less positive views about the scheme:

“*if it’s all about benefitting your baby, I don’t think high street vouchers are perhaps the right thing, I think it should have been something like I don’t know, like passes to toddler groups or vouchers for perhaps you know like kiddi care or something*” (F14)

These views highlight the importance of accurate information for eligible women and also that women who plan to breastfeed can feel disappointed when this does not happen, not merely because they could not apply for the scheme, but because their expectations regarding feeding their infant were not fulfilled.

### Perceived impact of the NOSH scheme

As part of the analysis, and in the light of positive trial results, we wanted to explore the impact of the NOSH scheme on infant feeding. This included the impact on decision making and the impact on breastfeeding continuation.

#### Impact on decision making

A view that had been expressed in the press was that the NOSH financial incentive amounted to “bribery” [16, 17] for breastfeeding. None of the participants in this study put forward the view (though the interviewers did not raise this topic – see Additional File A). Conversely, many interviewees reported that they had already decided to breastfeed before hearing about the scheme and women generally thought that vouchers would not make a difference for mothers who had strong opinions against breastfeeding, or for whom breastfeeding was not regarded as the norm or perceived as distasteful or even taboo:

*“if someone’s of the definitely of the other mind set, that they’re just not going to breast feed, I’m not sure that something like that would sway them, to be honest”* (T10)

However, some women suggested that participating in the scheme could provide a platform from which to raise the topic of, and normalise breastfeeding in social spheres, rejecting the social norms and negative discourses around breastfeeding (i.e. breastfeeding as “abnormal”) that they might encounter. This could eventually erode the idea that breastfeeding is no longer an option worth considering and open up more choices:

“*But, I think, what surprised me, like I don’t know with regards to my brother’s partner, I didn’t think that she’d breast feed at all and I don’t know whether me talking about it, and having sort of a positive experience of breast feeding, whether that’s influenced her in to have a go herself as* well”.

None of the interviewees reported financial difficulty as a reason for taking up the offer of the scheme, although many appreciated the impact that vouchers had on their monthly income at a time when expenses were increasing:

“*We still couldn’t believe that we got, money for, you know what I mean for food and everything, it really helped with the shopping. It really was amazing, it really, really was*” (T11)

Only one participant described having been at least partially influenced by the NOSH scheme to initiate breastfeeding:

“*It gave me that little extra incentive to at least try it and I kept, on that day when it felt like I couldn’t carry on I did keep pushing and pushing myself but in the end I was just like, couldn’t do it*” (T6)

The trial results show that rather than breastfeeding initiation being increased in intervention areas (which could be expected if women felt they were being “bribed” to breastfeed), it was the breastfeeding data at 6–8 weeks that showed a statistically significant increase. The following section describes how women described the impact of the scheme on breastfeeding continuation.

#### Help to keep going, reaching milestones

The timing of offered vouchers (2 days, 10 days, 6–8 weeks, 3 months and 6 months) was viewed positively by participating women. The anticipation of receiving vouchers, either as an extrinsic reward in the form of a “treat” or intrinsic reward such as a sense of feeling valued, was reported as an incentive to continue breastfeeding through difficult stages:

“*I wanted to breastfeed anyway but I think it has helped me with the various stages, I think I struggled at nine ten weeks and then knowing that I could keep going to twelve weeks it did encourage me. I don’t know so much if it was the vouchers that were coming I think it was just more the, you know the kind of three more weeks to go and it’s twelve weeks…*” (F18)

“*it does give you a little bit of extra incentive and when you reach each sort of milestone it does make you feel, sort of good about yourself, you’ve got to the next one*” (T11)

This was particularly pertinent at times when women were facing the wide range of physical, practical and socio-cultural challenges described in this article. Women reported that their early experiences of multiple feeds during the night were especially dispiriting and that the prospect of receiving vouchers would encourage them to continue and feel more positive than they might otherwise have done.

#### Compensating efforts: “Reward, bonus and boost”

For women who had already made a decision to breastfeed prior to becoming involved in the scheme, receiving vouchers was described in terms of “reward”, “bonus”, “boost”, a way of feeling valued and recognised for doing something positive for their baby.

“*It was like a little extra boost to think, you know what I mean, you’re doing something good, you’re doing the right thing and it’s helping. So, I appreciated that*” (T10)

Despite having strong preferences to breastfeed, a number of women described their experiences as anything but straightforward. Choosing to formula feed might have lessened the physical problems they faced, but receiving vouchers went some way toward compensating women when they managed to overcome difficulties, and gave them something to look forward to.

“*I think you feel almost like valued that you’re breastfeeding I think is how it felt and encouraged. I think the vouchers do then help as well*.” (F18)

These accounts highlight the complex and shifting decisions and experiences that women face regarding infant feeding, and the positive impact of the financial incentive in encouraging the continuation of breastfeeding once initiated.

## Discussion

These interviews need to be interpreted in the light of the NOSH trial results where there was evidence that the scheme was both acceptable (46% of all eligible mother-infant dyads were signed up for the scheme, and 34% claimed vouchers) and effective in increasing breastfeeding rates at 6–8 weeks in areas with low breastfeeding rates [12]. In this study, both participants and non-participants in the NOSH scheme described their experiences as mainly positive. However, non-participants reported a lack of clarity regarding eligibility for the scheme or not being able to apply for the scheme due to early problems.

Data from interviews suggest that although offering vouchers might (hypothetically) incentivise breastfeeding initiation, women generally attributed their initial infant feeding decisions to other influences. These findings support the idea that practical and health related factors are taken into account when weighing up feeding options prior to the birth [18]. They are also in line with evidence that pre and post birth decisions regarding infant feeding are influenced by a range of historical, socio-economic, cultural and individual factors [3]. Our findings show that decisions tend to be made in response to past experiences and support from others, as well as to social norms, although some women are able to overcome strong influences from well-meaning friends and family members if they are keen to breastfeed. Equally, women remain susceptible to the unpredictable effects of physical problems, lack of support and their own changing attitude to feeding following the birth. Giving up breastfeeding due to these effects can lead to feelings of guilt for women if they are not supported in having realistic expectations [19].

For women who decide to breastfeed, national statistics for England illustrate a steep decline in rates from initiation (73.8%) to breastfeeding rates at 6–8 weeks (45.2%) in the first quarter of 2015–2016 [5], suggesting that it is the continuation of breastfeeding that women find challenging. Continuation is reported to be more likely where women intend to breastfeed and are positive about breastfeeding [20]. Our findings show that even when women are positive about breastfeeding, they often face a range of difficulties that can discourage them from continuing. Financial incentives did seem to help women to work through some of the more personal difficulties associated with breastfeeding and also to motivate them to continue to breastfeed to reach the more general milestones at which the vouchers were offered. Women who received vouchers regarded them as a reward, a bonus and a boost for the challenges they faced during breastfeeding and felt valued for their efforts:

“*it added the incentive to carry on and it sort of gives you a little boost of help, especially in the early months it’s very important to have enough money to eat well*” (T11)

These findings resonate with those of Thomson et al. [7] who reported that women who breastfed felt rewarded by the gift they received from an incentive scheme.

Women in our study also suggested ways that the scheme might indirectly impact on future infant feeding decisions, including changing the social norm around breastfeeding. A number of women associated low breastfeeding rates with cultural and social circumstances, and negative discourses of breastfeeding. Raising awareness of breastfeeding and treating it as “normal” was one way that they thought the scheme might help to break through these discourses and gradually increase breastfeeding rates. The concept of ‘normalcy’ derived from breastfeeding support has previously been identified where mothers faced embarrassment and isolation in a culture that demeaned breastfeeding, particularly in public [21]. Rollins et al. [3] support the need for positive messages around breastfeeding in society, as well as practical changes that enable women to breastfeed without disadvantage and stigma, for example, ensuring that maternity leave and public space policies are breastfeeding friendly.

### Strengths and limitations

This is the first study of women’s experiences of being offered a financial incentive to breastfeed. It was conducted alongside a large cluster randomised controlled trial of the financial incentive (details [11] and findings [12] are published elsewhere) to explore acceptability of the scheme in practice and to contextualise the trial findings [22].

All attempts were made to include women with a range of perspectives by targeting first time and experienced mothers from different age groups living in a range of IMD (Index of Multiple Deprivation) profiles. Despite these efforts the findings may not be fully representative of all women who took part in the NOSH scheme. Women who came forward to be interviewed were mostly older and from the least deprived areas. It could be argued that older and more affluent women might be more likely to breastfeed [23] and to participate in interviews. Women were also of white ethnicity and nearly all were British, which limits generalisation of the findings to diverse populations. However, non-white women are generally associated with higher rates of breastfeeding [23] and therefore the study was not specifically examining the influence of ethnicity on breastfeeding or participation in the scheme.

New mothers are busy and occasionally had to cancel interviews. Nevertheless, the views presented here are helpful in determining how the scheme was received, the perceived impact of the intervention and some potential ways that the scheme might impact on decision-making. We consider that the experiential backgrounds of the interviewers helped them to conduct the interviews in a sensitive yet conversational style whilst adhering to the research objectives.

## Conclusions

A range of practical and socio-cultural factors influence infant feeding decisions at important stages in the process. Talking about the scheme with others provided a channel through which negative beliefs about breastfeeding could be rejected. Women described the vouchers as a ‘reward’ which incentivised continued feeding. These suggest possible mechanisms of action for the statistically significant increase in breastfeeding at 6–8 weeks reported in the trial of the NOSH Scheme.

These interviews with participants gave no credence to initial media-reported claims that a financial incentive scheme amounted to “bribery” to breastfeed [16, 17]. These interviews appear to support the trial findings that the scheme had a greater impact on the decision to continue breastfeeding for longer, rather than on the decision to initiate breastfeeding.

## Additional files

Additional file 1: Topic Guides. (DOCX 14 kb)

Additional file 2: Framework matrices. (DOCX 15 kb)

## Abbreviations

HCP: Health Care Professional
IMD: Index of Multiple Deprivation
NOSH: Nourishing Start for Health
PhD: Doctor of Philosophy
UK: United Kingdom
WHO: World Health Organization
