# Supplementary material for: Valuing breastfeeding: a qualitative study of women’s experiences of a financial incentive scheme for breastfeeding
Source: BMC Pregnancy Childbirth. 2018 Jan 8;18:20. doi: 10.1186/s12884-017-1651-7 (PMC5759235; doi:10.1186/s12884-017-1651-7)
Supplement: Supplementary file 1 — Topic Guides. (DOCX 14 kb) [file 12884_2017_1651_MOESM1_ESM.docx]

**Additional file 1: Topic Guides**

***Women who sent in NOSH claim forms***

**Details on hearing about the scheme**

o How they heard about the NOSH scheme?

o What their initial thoughts were?

**Information obtained about breastfeeding antenatally**

o Separate to the NOSH scheme what other information did they get about breastfeeding?

o Other influences on decision to breastfeed?

**Experience of applying for the scheme**

o Steps involved in applying to join the NOSH scheme including the Welcome Pack?

o Thoughts on this process?

**Experience in maternity hospital**

o Experience of starting feeding baby?

o Discussion of scheme in maternity hospital?

**Experience once discharged from maternity hospital and signing of claim forms**

o Being discharged from maternity unit and support for breastfeeding?

o Experience with getting claim forms signed at different time points?

o Experience of breastfeeding?

o Views on claiming vouchers from NOSH Office?

**General questions about the scheme**

o Views on the scheme and how it worked?

o Did the NOSH scheme encourage breastfeeding for longer?

o Any suggested changes to the scheme?

o Any additional comments?

***Women who applied for the NOSH Scheme but did not send in claim forms***

**Details on hearing about the scheme**

o How they heard about the scheme?

o What their initial thoughts were?

**Information obtained about breastfeeding antenatally**

o Separate to the scheme what other information did they get about breastfeeding?

o Any other influences on decision to breastfeed?

**Experience of applying for the scheme**

o Steps involved in applying to participate for the NOSH scheme?

o Thoughts on this process?

o Receiving the welcome pack and thoughts on this?

**Experience in maternity hospital**

o Experience of starting feeding baby?

o Discussion of scheme in maternity hospital?

**Experience once discharged from maternity hospital**

o Experience at home once discharged from maternity unit?

o At what stage was decision made not to participate in the scheme and why?

**Final questions**

o In light of experience views on the scheme?

o Any suggested changes to the scheme?

o Any additional comments?

***Eligible women who did not apply for the NOSH Scheme***

**Details about decision on choice of infant feeding method**

o Influences on decision on how to feed baby?

o When decision was made?

**Information about whether they heard about the NOSH scheme**

o Did they hear about the NOSH scheme? If no, why do they think this was? If yes, how and their initial views on the scheme?

**Experience of infant feeding**

o Experience of feeding baby?
