# Supplementary material for: Valuing breastfeeding: a qualitative study of women’s experiences of a financial incentive scheme for breastfeeding
Source: BMC Pregnancy Childbirth. 2018 Jan 8;18:20. doi: 10.1186/s12884-017-1651-7 (PMC5759235; doi:10.1186/s12884-017-1651-7)
Supplement: Supplementary file 2 — Framework matrices. (DOCX 15 kb) [file 12884_2017_1651_MOESM2_ESM.docx]

**Additional file 2: Themes from Framework analysis**

| **Feasibility Phase** | | |
| --- | --- | --- |
| **Antenatal decision regarding infant feeding** | Already decided to breastfeed |  |
|  | Reluctant decision to try to breastfeed |  |
|  | Decision to mix feed |  |
|  | Decided not to breastfeed then tried it |  |
| **Experience of breastfeeding** | Past or present experiences | *Positive experiences*  *Difficult experiences*  *Level of support* |
| **Hearing about and discussing the scheme** | HCPs | *Level of information from HCP* |
|  | Media, social media, publicity |  |
|  | When heard about scheme | *Pregnancy*  *After birth* |
|  | Research / NOSH website |  |
|  | Perception / understanding of scheme |  |
| **Discussing the scheme with family and friends** | Supportive reaction |  |
|  | Negative reaction |  |
|  | Mother able to ignore other’s views |  |
|  | Neutral / mixed reaction or general discussion |  |
|  | Prevented from applying and breastfeeding, not from choice |  |
| **Influences on infant feeding decision** | NOSH influence on initial decision | *No influence*  *Potential influence*  *Contradiction of views* |
|  | NOSH influence on breastfeeding continuation |  |
|  | Other influences on infant feeding method |  |
|  | Influences on changing infant feeding method |  |
| **Reasons for participation / non-participation in the scheme** | Previous experience  Incentive  Hearing about the scheme  Influence of others  Reward for breastfeeding |  |
| **Positive views of the scheme** | Views that are theoretical |  |
|  | Views based on experience |  |
| **Suggested changes to scheme** |  |  |
| **Trial Phase** | | |
| **Experiences of infant feeding** | Decision regarding feeding |  |
|  | Culture |  |
|  | Impact of scheme on decision |  |
|  | Knowledge about infant feeding |  |
|  | Previous children |  |
|  | Support from family and friends |  |
|  | Support from HCPs | *Ante-natal support* |
|  |  | *Maternity Ward Support* |
|  |  | *Post-natal support* |
|  | Experience of feeding |  |
| **Views of the NOSH scheme** | Negative views of the scheme |  |
|  | Positive views of the scheme |  |
| **Experiences of the NOSH scheme** | Applying for the scheme | *Hearing about the scheme* |
|  |  | *Discussing scheme with others* |
|  | Participation | *Claim forms* |
|  |  | *Receiving the Welcome pack* |
| **Rolling out the scheme** | Suggested changes to the scheme |  |
|  | Suggested research |  |
